# Acute Effects of Three Neuromuscular Warm-Up Strategies on Several Physical Performance Measures in Football Players

**DOI:** 10.1371/journal.pone.0169660

**Published:** 2017-01-06

**Authors:** Francisco Ayala, Ana Calderón-López, Juan Carlos Delgado-Gosálbez, Sergio Parra-Sánchez, Carlos Pomares-Noguera, Sergio Hernández-Sánchez, Alejandro López-Valenciano, Mark De Ste Croix

**Affiliations:** 1 Sports Research Centre, Miguel Hernández University of Elche, Alicante, Spain; 2 Department of Pathology and Surgery, Physiotherapy Area, Miguel Hernández University of Elche, Alicante, Spain; 3 School of Physical Education, Faculty of Sport, Health and Social Care, University of Gloucestershire, Gloucester, United Kingdom; Victoria University, AUSTRALIA

## Abstract

No studies have analysed the acute effects of the FIFA 11+ and Harmoknee warm-up programmes on major physical performance measures. The aim of this study was to analyse the acute (post-exercise) effects of the FIFA 11+, Harmoknee and dynamic warm-up routines on several physical performance measures in amateur football players. A randomized, crossover and counterbalanced study design was used to address the purpose of this study. A total of sixteen amateur football players completed the following protocols in a randomized order on separate days: a) FIFA 11+; b) Harmoknee; and c) dynamic warm-up (DWU). In each experimental session, 19 physical performance measures (joint range of motion, hamstring to quadriceps [H/Q] strength ratios, dynamic postural control, 10 and 20 m sprint times, jump height and reactive strength index) were assessed. Measures were compared via a magnitude-based inference analysis. The results of this study showed no main effects between paired comparisons (FIFA 11+ vs. DWU, Harmoknee vs. DWU and Harmoknee vs. FIFA 11+) for joint range of motions, dynamic postural control, H/Q ratios, jumping height and reactive strength index measures. However, significant main effects (likely effects with a probability of >75–99%) were found for 10 (1.7%) and 20 (2.4%) m sprint times, demonstrating that both the FIFA 11+ and Harmoknee resulted in slower sprint times in comparison with the DWU. Therefore, neither the FIFA 11+ nor the Harmoknee routines appear to be preferable to dynamic warm-up routines currently performed by most football players prior to training sessions and matches.

## Introduction

The FIFA 11+ [1] and Harmoknee [2] are two warm-up programmes designed to prevent and reduce the number and severity of football-related injuries, particularly in amateur players. Both programmes include running exercises and specific dynamic movements focusing on the major neuromuscular risk factors for lower extremity injuries (i.e. leg strength, balance, dynamic postural control, agility, knee control during cutting and landing, joint range of motion), based on scientific evidence and best practice [1, 2]. In addition to the running and neuromuscular exercises, the FIFA 11+ and Harmoknee also include sprinting, multidirectional speed and plyometric exercises.

Recent cluster randomized controlled trials have demonstrated that the FIFA 11+ is effective in reducing lower extremity injury rates (mainly knee injuries) in teams practising this warm-up at least twice a week for longer than three consecutive months [3–8]. Although with less rigour than the FIFA 11+, the effectiveness of the Harmoknee to reduce the incidence of lower extremity injuries (mainly knee injuries) has also been documented [2]. The potential training (chronic) effects behind the reported reduction in injury incidence produced by the FIFA 11+ and Harmoknee appear to be related to improvement in neuromuscular control of the trunk and lower extremities [9–11]. These positive results in injury incidence rates in combination with the fact that no additional or specific equipment (for example balance boards) is required have led some research groups and institutions to develop countrywide campaigns to implement the FIFA 11+ and Harmoknee in the everyday football training routines (especially at amateur levels) [12, 13].

It is widely accepted that a “good” or “appropriate” warm-up programme should be able to improve performance (via post activation potentiation, decrease in stiffness, rise in core temperature and resting oxygen consumption [14]) but should not be to demanding to cause detrimental effects due to fatigue related factors [15]. Therefore, both, the FIFA 11+ and Harmoknee must demonstrate that they are able to elicit physiological acute changes (post-exercise effects) to positively affect the major physical performance measures (i.e. sprinting, jumping, range of motion, etc.) before being considered as appropriate warm-up programmes to be performed prior to formal training and competition. Furthermore, if the use of the FIFA 11+ and/or Harmoknee is to be promoted at the expense of dynamic warm-up programmes currently performed by most amateur football teams, the magnitude of their hypothetical positive effects on physical performance measures should be at least similar to those reported by the latter mentioned warm-up programmes [16–24]. Otherwise, the FIFA 11+ and Harmoknee should be implemented in everyday football sessions as training components (i.e. placed in the main part of the training sessions), based on their demonstrated superior positive effects on injury incidence rates in comparison with traditional practices, rather than being used as a pre-exercise warm-up routine.

However, analysing the body of literature regarding the acute effects of the FIFA 11+ and Harmoknee on sports performance, a smaller evidence base supporting their use as "good warm-up programmes" appears to be available in comparison with the number of studies that have documented their efficacy as long-term interventions to reduce the injury incidence [3–8, 12, 13]. To the best of our knowledge, only Bizzini et al. [15] have examined the pre-exercise effects of the FIFA 11+ on various sports-performance variables, showing improvements in 20 m sprint time, jump height and agility comparable with those obtained with other dynamic warm-up routines reported in the literature. In addition, Bizzini et al. [15] reported that the FIFA 11+ induced similar improvement in resting oxygen uptake, core temperature and lactate as those obtained with other warm-up routines. However, no studies (to the authors´ knowledge) have analysed the acute effects of the Harmoknee on physical performance measures.

Therefore, the main purpose of this study was to analyse the acute (post-exercise) effects of the FIFA 11+, Harmoknee and dynamic warm-up routines on several physical performance measures in amateur football players. We hypothesize that there would not be significant differences in the acute effects elicited by the three football-related warm-up protocols selected on the physical performance measures analysed based on the fact that the duration (approximately 25 min) and type of exercises (neuromuscular exercises) in each routine are similar.

## Materials and Methods

### Design

A randomized, post-test only crossover and counterbalanced study design, in which participants performed all experimental conditions, was used to address the aims of this study. The use of a pre- and post-test crossover design, in which participants performed a pre- and post-warm up physical performance assessment was not adopted because in a pilot study (n = 4 males and 2 females) the participants consistently reported that the pre and post warm-up assessment procedure used was too long and subsequently they felt less able or fatigued to undertake the post-warm up assessment and hence, bias the results.

The independent variables were the three different intervention routines (FIFA 11+, Harmoknee and dynamic warm-up). The dependent variables included 19 physical performance measures (range of motion, dynamic postural control, conventional and functional hamstring-to-quadriceps strength ratios, 10 and 20 m sprint times, jumping height and reactive strength index).

Participants visited our laboratory on four occasions, with a week’s rest interval between sessions. Each testing session was carried out 48–72 hours after finishing the previous competitive match (i.e. Tuesday or Wednesday) so that the players could have enough time for recovery. Furthermore, players did not carry out any training session throughout this rest-interval. To minimize circadian variation and other similar effects on physical performance, each participant carried out all experimental sessions at the same time of day (in the late afternoon or early evening, depending on the participants availability) and under the same environmental conditions (room temperature at 25°C).

The first visit was a practice/habituation session to the different testing procedures and warm-up exercises, and the following three visits were the experimental sessions.

During each experimental session, participants began by completing one of the three interventions: the FIFA 11+, the Harmoknee or the dynamic warm-up. The order of interventions was randomised per person using a computer-based software programme (www.randomiser.org) to avoid carry-over effects. The assessment of the physical performance measures was carried out 2–3 minutes (post-test) after the entire warm-up programme was completed. This time elapse between the end of the warm-up and the start of the assessment of the physical performance measures was selected because: a) it reflects the typical period of time existing between the end of the warm-up and the start of a match in the amateur leagues; and b) to be consistent with similar previous studies [15, 22].

**Fig 1:**
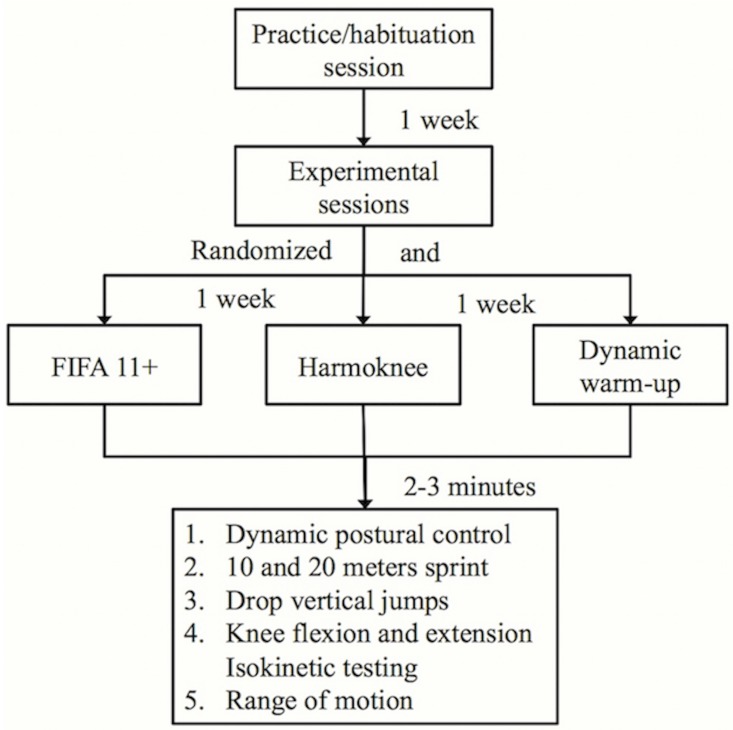
Schematic representation of the study design.

The order of the tests was consistent throughout the experimental sessions and was established with the intention of minimizing any possible negative influence among variables (Fig 1). The mean duration of each experimental session was 65.3 ± 8.6 min. A priori, the overall duration of the post-warm up physical performance assessment (45 min approximately) carried out within each experimental session, although similar to previous studies [15, 20, 24–26], could appear to be enough to generate fatigue in the participants to bias the results (at least in the latter testing measures). However, the above-mentioned pilot study also showed that the order of the tests, the number of trials performed, as well as the rest intervals within trials and among tests were sufficient in order not to produce meaningful musculoskeletal fatigue in the participants. Furthermore, a “damping phenomenon” (i.e. the physical demands required by the test battery may offset the potential effects of an intervention) was not expected due to the fact that the sport-specific dynamic movement components of the three warm ups included activities with very similar neuromuscular and energetic requirements to those needed to successfully perform in the tests. In addition, Ayala et al. [25] reported that the time-course of the effects elicited by a dynamic warm up with similar characteristics (duration, type of exercises, intensity) to those used in the current study on some physical performance measures (i.e. 10 and 20 m sprint, jumping height) lasted more than 30 minutes while participants engaged in a simulated tennis match. Therefore, a decline in the magnitude of the effects elicited by the three interventions on physical performance throughout the length of the post-warm up assessments was not expected.

Each experimental session was carried out under the strict supervision of the researchers.

### Participants

Twenty-two participants, consisting of 12 men and 10 women who were amateur football players, took part in the current study. Participants belonged to five different football teams that were engaged in the Official Amateur Championships of the Spanish Football Federation (fourth [regional] Spanish division). Although all participants reported taking part in regional football leagues, none was involved in a systematic and specific strength-training programme. Likewise, their training frequency and duration through the study was 3 days per week, 1.5 hours per session. To reduce the interference of uncontrolled variables, all the participants were instructed to maintain their habitual lifestyle and normal dietary intake before and during the study. The participants were told not to exercise on the day before a test and to consume their last (caffeine-free) meal at least 3 hours before the scheduled test time.

Other exclusion criteria were: (1) histories of neuromuscular diseases or musculoskeletal injuries specific to the shoulder, ankle, knee, or hip joints over the past 6 months; (2) missing one testing session during the data collection phase; (3) and presence of self-reported delayed onset muscle soreness at any testing session. Before any participation, experimental procedures and potential risks were explained fully to the participants both verbally and in writing. Written informed consent was obtained from the players. The Institutional Research Ethics Committee approved the study participant information sheet, design and testing protocols prior data collection.

Eight men (age = 19.1 ± 1.3 y; stature = 177.2 ± 6.4 cm; body mass = 71.4 ± 8.8 kg) and eight women (age = 20.1 ± 1.8 y; stature = 164.9 ± 6.9 cm; body mass = 60.1 ± 6.6 kg) classified as amateur football players completed this study. Four men and two women were excluded from the study because they missed one or more of the testing sessions.

### Interventions

#### FIFA 11+

The FIFA 11+ consisted of three parts (Table 1), the first of which involved running exercises (part 1). The second part covered six exercises, all of which comprised three levels of difficulty and were aimed at improving strength, balance, muscle control and core stability (part 2). The third and final part consisted of advanced running exercises (part 3). All players were able to perform level II of difficulty for each exercise in part 2, which was confirmed during the familiarization session. However, no player was able to perform the level III of difficulty for each exercise. Therefore, the level II of difficulty was chosen for this study.

**Table 1:** FIFA 11+ warm-up programme*.

Exercise	Duration
Part 1: Running exercises	8 minutes
1. Straight ahead	2 sets over 30 m each exercise
2. Hip out	
3. Hip in	
4. Circling partner	
5. Shoulder contact	
6. Quick forward & backwards	
Part 2: Strength—Plyometric—Balance	10 minutes
7. The bench: alternate legs	3 sets x 40 s (lifting 2 s each leg in turn)
8. Sideways bench: raise and lower hip	3 sets x 20 repetitions each side
9. Hamstrings: intermediate	1 set x 7 repetitions
10. Single-leg stance: throwing ball with partner	2 set x 30 s each leg
11. Squats: walking lunges	2 set x 10 repetitions each leg
12. Jumping: lateral jumps	2 set x 15 jumps (30 s approximately)
Part 3: Running exercises	2 minutes
13. Across the pitch	2 sets x 30 m (70–80% maximum pace)
14. Bounding	2 sets x 30 m
15. Plant and cut	2 sets x 5 repetitions (80–90% maximum pace)

m: meters, s: seconds;

*: for more details see the manual and instructions freely available on the official website www.f-marc.com/11plus.

#### Harmoknee

The Harmoknee warm-up programme included 5 parts: warm-up, muscle activation, balance, strength and core stability, all of which can be combined and performed in a regular soccer training session (Table 2). Total programme duration was 20 to 25 minutes.

**Table 2:** Harmoknee warm up programme*.

Exercise	Duration
Part 1: Warm up	10 minutes
1. Jogging	4 minutes
2. Backward jogging on the toes	1 minute
3. High-knee skipping	30 s
4. Defensive pressure technique	30 s
5. One and one	2 minutes
Part 2: Muscle activation	2 minutes
6. Calf	4 s each leg/side
7. Quadriceps	
8. Hamstrings	
9. Hip flexor muscles	
10. Groin muscles	
11. Hip and lower back muscles	
Part 3: Balance	2 minutes
12. Forward and backward double leg jumps	30 s
13. Lateral single leg jumps	
14. Forward and backward single leg jumps	
15. Double leg jump with or without ball	
Part 4: Strength	4 minutes (1 min each exercise)
16. Walking lunges in place	15 repetitions each leg
17. Hamstring curl	12 repetitions
18. Single-knee squat with toe raises	12 repetitions
Part 5: Core stability	4 minutes (1 min each exercise)
19. Sit-ups	2 sets x 12 repetitions
20. Plank on elbows and toes	2 sets x 20 s
21. Bridging	2 sets x 12 repetitions

s: seconds;

*: for more details see http://www.harmoknee.com

#### Dynamic warm-up

Ten different cards showing dynamic warm-up routines were given to each participant. Seven of the dynamic warm-up routines were extracted from the studies selected in the meta-analysis carried out by Bizzini et al. [15] in the context of the acute effects of dynamic warm-ups [18–21, 24, 27, 28]. The other three warm-up routines were selected because they reflect the warm-up structure and content that, according to our experience, might be the most widely used in football and which contain the following components chronologically ordered: a) some active aerobic activities (including running, light calisthenics); b) dynamic stretching exercises of the major muscle groups; and c) football-specific movements incorporating a various range of motion exercises with skill-based drills executed at, or just below game intensity [22, 29, 30]. Those published warm-up routines that required additional equipment (for example, balance boards, dumb-bells, weight vests or barbells) were not considered for the purpose of this study because they do not represent the reality of most amateur clubs and teams. After that, each participant picked up the three cards that most closely replicated his/her habitual warm-up routine. Three points were given to the most closely related warm-up routine, two points to the second and one point to the last warm-up routine. The dynamic warm-up routine considered the ‘standard’ must have been picked up and scores with two or three points by at least 70% of the participants. In the case of more than one dynamic warm-up routine meeting the above-mentioned criterion, the one with the highest summation of points was selected as standard. Two dynamic warm-up routines were picked up by at least 70% of the participants [22, 29], these being the dynamic warm-up routine designed by Taylor et al. [22], which reached the highest score and hence was considered as the standard football-related warm-up routine (Table 3).

**Table 3:** Dynamic warm up programme*.

Exercise	Duration
1. High knees	3 set over 20 m
2. Butt flicks	3 set over 20 m
3. Carioca	3 set over 20 m each side
4. Dynamic hamstring swings	10 repetitions each leg
5. Dynamic groin swings	10 repetitions each leg
6. Arm swings: forwards and backwards	10 repetitions each direction
7. Faster high knees (shorter stride)	4 sets over 10 m
8. Swerving	2 sets over 30 m at 70% of maximum pace
9. Side stepping	2 sets over 30 m at 80% of maximum pace
10. Spiderman walks	1 set over 20 m
11. Sideways low squat walks	1 set x 10 steps each direction
12. Upper body rotations	10 repetitions each leg
13. Vertical jump	5 repetitions building in intensity
14. Run through	2 sets x 20 m at 70% of maximum pace 2 sets x 20 m at 80% of maximum pace 1 set x 20 m at 90% of maximum pace
15. Countermovement jump then 5 m sprint	2 sets x 5 m at 90% of maximum pace 1 sets x 5 m at 95% of maximum pace
16. Sprint for 5 m then countermovement jump	2 sets x 5 m

m: meters;

*: warm up programme extracted from Taylor et al. [22]

### Physical performance measures

#### Hip, knee and ankle range of motions

The passive hip flexion (passive straight leg raise test [Fig a in S1 appendix]), extension (Thomas test [Fig b in S1 appendix]) and abduction (passive hip abduction with knee flexed over the edge of the plinth test [Fig c in S1 appendix]); knee flexion (Modified Thomas test [Fig d in S1 appendix]) and ankle dorsiflexion (weight-bearing lunge with knee extended [Fig e in S1 appendix] and flexed [Fig f in S1 appendix] tests) range of motions of the dominant and non-dominant extremities were assessed following the methodology previously described [31]. Participants were instructed to perform, in a randomized order, two maximal trials of each range of motion test for each extremity. The mean score for each test was used in the subsequent analyses. The same researchers performed the ROM testing at all testing sessions.

#### Conventional and functional hamstring-to-quadriceps strength ratios

The assessment of the H/Q_CONV_ and H/Q_FUNCT_ strength ratios was carried out following the methodology described by Ayala et al. [32]. Briefly, a Biodex System-4 isokinetic dynamometer (Biodex Corp., Shirley, NY, USA) and its respective manufacture software were used to determine isokinetic concentric and eccentric torques during knee extension and flexion actions. Only the dominant leg was tested, as no meaningful differences between legs have been previously reported for sedentary and recreationally active adults [33, 34]. The dynamometer was calibrated according to the manufacturer’s instructions immediately before each test session and verified immediately after to ensure that no changes occurred in sensitivity.

Participants were secured supine on the dynamometer with the hip passively flexed at 10°–20° (S2 appendix). The axis of rotation of the dynamometer lever arm was aligned with the lateral epicondyle of the knee. The force pad was placed approximately 3 cm superior to the medial malleolus, with the foot in a relaxed position. Adjustable strapping across the pelvis, thigh proximal to the knee and foot localized the action of the musculature involved. The range of movement was set from 90° knee flexion (starting position) to 0° (0° was determined as maximal voluntary knee extension for each participant).

Before isokinetic testing, the participants performed a specific isokinetic warm-up consisting of three sub-maximal (self-perceived 50% effort) and two maximal concentric and eccentric knee extension and flexion actions at 120°/s.

The isokinetic examination was separated into two parts. The first part of the examination was the assessment of the knee extensor and knee flexor muscles during concentric/concentric (CON/CON) cycles with extension undertaken first. After a 5 min rest period the eccentric/eccentric (ECC/ECC) testing cycle was performed.

In both testing methods, three cycles of knee flexions and extensions were performed at three preset constant angular velocities in the following order: 60, 180 and 240°/s for CON/CON cycles; and 30, 60 and 180°/s for ECC/ECC cycles (slow to fast). The two testing parts (CON/CON and ECC/ECC) were separated by a 5 min rest interval and a rest of 30 s was allowed between action cycles. The number of maximal muscle actions and the rest period durations were chosen to minimize musculoskeletal fatigue, which is unlikely to occur (based on the participants’ perceptions reported in the pilot study) with only three muscle actions at three velocities and 30 s rest between muscle actions and velocities and 5 min rest between testing modes (concentric and eccentric). For both concentric and eccentric actions, participants were encouraged to push–pull/resist as hard and as fast as possible and to complete the full range of motion. Participants were instructed to abort the test if they felt any discomfort or pain. During the test, all participants were given visual feedback from the system monitor. They were also verbally encouraged by the investigator to give their maximal effort, and the instructions were standardized by using key words such as ‘resist’, ‘push’ and ‘hard and fast as possible’.

The peak torque was extracted for each of the three trials performed at each velocity during the CON/CON and ECC/ECC cycles of reciprocal knee extension and flexion movements through the three testing sessions. For each isokinetic peak torque variable, the average of the three trials at each velocity was used for the subsequent calculation of the conventional and functional hamstring-to-quadriceps strength ratios due to the magnitude of the error component decreasing with increased trials [35].

Thus, the H/Q_CONV_ ratios were calculated as the ratio between the torques produced concentrically by hamstrings and quadriceps during the isokinetic tests at 60 (H/Q_CONV60_) and 180°/s (H/Q_CONV180_). H/Q_FUNC_ ratios were calculated as the ratio between the torques produced eccentrically by the hamstring and concentrically by the quadriceps muscles at 60 (H/Q_FUNC60_) and 180°/s (H/Q_FUNC180_). Finally, the H/Q_FUNC_ proposed by Croisier et al. [36] was also calculated as the ratio between the torques produced eccentrically by the hamstring at 30°/s and concentrically by the quadriceps muscles at 240°/s (H/Q_FUNC30/240_).

#### Dynamic postural control

Dynamic postural control was evaluated using the Y-Balance test and following the guidelines proposed by Shaffer et al. [37]. Players were allowed a maximum of five trials to obtain three successful trials for each reach direction (anterior [Fig a in S3 appendix], posteromedial [Fig b in S3 appendix] and posterolateral [Fig c in S3 appendix]). Trials were discarded if the player failed to maintain unilateral stance on the platform, failed to maintain reach foot contact with the reach indicator on the target area while the reach indicator is in motion, used the reach indicator for stance support, or failed to return the reach foot to the starting position under control [37]. Specifically, the testing order was completed as dominant anterior, non-dominant anterior, dominant posteromedial, non-dominant posteromedial, dominant posterolateral, and non-dominant posterolateral. The average of the three reaches was normalized by dividing by the previously measured leg length to standardize the maximum reach distance ([excursion distance/leg length] x100 = % maximum reach distance) [38]. Leg length was defined as the length measured in centimetres from the anterior superior iliac spine (ASIS) to the most distal portion of the medial tibial malleolus. To obtain a global measure of the balance test, data from each direction were averaged for calculating a composite score [39].

#### 10 and 20 meters sprint time

Owing to their good reproducibility, linear sprint tests ranging from 10 to 20 m are used as general measures of linear acceleration and speed in football players. Time during a 20 m sprint in a straight line was measured by means of single beam photocell gates placed 1.0 m above the ground level (Time It; Eleiko Sport, Halmstad, Sweden). Each sprint was initiated from an individually chosen standing position, 50 cm behind the photocell gate, which started a digital timer. Each player performed three maximal 20 m sprints interspersed with 1 min of passive recovery, and the mean of the two fastest times achieved was retained.

#### Drop vertical jump height

A vertical drop jump (DJ) without arm swing was performed on a force platform (Kistler, Switzerland) according to Onate et al. [40]. Participants stood with feet shoulder-width apart on a 28 cm high step, 30 cm from the contact platform. They were instructed to lean forward and drop from the step as vertically as possible, in an attempt to standardize landing height. Participants were required to land with one foot on each of the force plates, then immediately perform a maximal vertical jump, finally landing back on the contact platform. Participants were asked to keep their hands on their hips to prevent the influence of arm movements on vertical jump performance. Each participant performed five maximal jumps starting from a standing position, with at least 30 s of recovery between jumps. Participants were asked to jump as high as possible. The mean jump height of the best three jumps was used for statistical analysis.

#### Reactive strength index

The reactive strength index was calculated using the data from the drop vertical jumps performed by the participants and following this formula [41, 42]:

Reactive strength index = Jumping height (mm)/ground contact time (ms)

The mean reactive strength index of the best three jumps was used for statistical analysis

### Statistical analysis

Descriptive statistics (means ± standard deviations) were calculated for all physical performance measures at post warm-up interventions.

Dependent sample t-tests were carried out to assess differences between limbs (dominant versus non-dominant) in dynamic postural control and ROMs.

Magnitude-based inferences of differences between-groups were calculated for each variable using a spreadsheet designed by Hopkins [43]. Each participant’s change score between paired sessions (dynamic warm-up vs. FIFA 11+; dynamic warm-up vs. Harmoknee; FIFA 11+ vs. Harmoknee) was expressed as a percentage of baseline score via analysis of log-transformed values, to reduce bias arising from non-uniformity of error. Errors of measurement and individual responses expressed as coefficients of variation were also estimated. In addition, the analysis determines the probability that the true effects are substantial or trivial when a value for the smallest substantial change is entered.

Coefficients of variation (CV) determined the smallest substantial change for each of the variables. The CV (standard error of measure [SEM] or typical error of measure [TE] expressed as percentage) data reported by previous inter-session reliability studies for each variable were used for the magnitude-based inference analyses. Thus, substantial is an absolute change > 2.5% for measures of range of motion [31], 18.5% for measures of H/Q strength ratios [32], 3.0% for measures of dynamic postural control [44], 1% for 10 and 20 m sprint times [45], 6% for DJ height [46] and 13.9% for measures of reactive strength index [47]. The qualitative descriptors proposed by Batterham and Hopkins [48] were used to interpret the probabilities (clinical inferences based on threshold chances of harm and benefit of 0.5% and 25%) that the true effects are harmful, trivial or beneficial: <1%, almost certainly not; 1–4%, very unlikely; 5–24%, unlikely or probably not; 25–74%, possibly or maybe; 75–94%, likely or probably; 95–99%, very likely; >99%, almost certainly. This approach to qualitatively describe the inferences is based on where the confidence interval of the between-groups differences lies in relation to a 3-level (beneficial, trivial and harmful) scale of magnitudes. For example, whether a confidence interval is entirely within the beneficial range of the smallest substantial change, the effect is clearly beneficial (>99%, almost certainly). Contrarily, if the confident interval spans 2 levels; harmful and trivial or trivial and beneficial, then the inference is qualified with a descriptor that represents the likelihood that the true value will have the observed magnitude (probabilistic inference) [49]. The inference was deemed unclear when the 90% confidence interval of the pre-post change differences overlapped both beneficial and harmful levels.

This spreadsheet also provides estimates of the effect of an intervention adjusted to any chosen value of a covariate, thereby reducing the possibility for confounding effects (e.g when a characteristic is unequal in the experimental and control groups). Thus, the sex of the participants was included as a covariate.

The current study considered a “substantial” main effect when a change was noted between paired-comparisons in physical performance measures that had reported a probability of the worthwhile differences of “likely” or higher (> 75% positive or negative).

## Results

The statistical analysis showed no significant differences (p values from 0.53 to 0.95) in joint range of motions (hip flexion, extension and abduction; knee flexion and ankle dorsiflexion with knee flexed and extended) and dynamic postural control (anterior, posteromedial and posterolateral directions) outcomes between the dominant and non-dominant limbs of the players. Consequently, the average score of both limbs for each unilateral variable was used for the subsequent statistical analysis. The post intervention results of variables are reported for descriptive purposes in Table 4.

**Table 4:** post-intervention (Dynamic warm-up, Harmoknee and FIFA 11+) results (mean ± standard deviation [SD]) for physical performance outcomes.

Variable	Dynamic warm-up	Harmoknee	FIFA 11+
Y-Balance test (cm)^T^			
• Anterior distance	64.9 ±5.7	63.5 ±4.3	63.6 ±5.1
• Posteromedial distance	101.7 ±8.6	103.9 ±7.8	104.4 ±8.4
• Posterolateral distance	96.8 ±7.4	95.4 ±6.1	97.2 ±7.8
• Composite	84.4 ±6.1	84.4 ±4.9	85.3 ±5.8
Sprint time (s)			
• 10 m	1.99 ±0.17	2.07 ±0.15	2.12 ±0.18
• 20 m	3.44 ±0.31	3.47 ±0.27	3.53 ±0.34
Vertical drop jump			
• Height (cm)	27.1 ±3.1	22.9 ±2.6	24.6 ±2.9
• Reactive strength index	1.04 ±0.29	1.07 ±0.27	1.05 ±0.28
Hamstring-to-quadriceps strength ratios			
• H/Q_CONV60_	0.60 ±0.06	0.60 ±0.07	0.59 ± 0.08
• H/Q_CONV180_	0.64 ±0.14	0.66 ±0.13	0.64 ±0.14
• H/Q_FUNCT60_	1.33 ±0.34	1.23 ±0.29	1.29 ±0.18
• H/Q_FUNCT180_	1.63 ±0.41	1.58 ±0.52	1.63 ±0.41
• H/Q_FUNCT30/240_	1.99 ±0.62	1.92 ±0.65	2.06 ±0.47
Joint range of motion (°)			
• Hip flexion	67.4 ±11.6	67.3 ±11.6	69.9 ±14.9
• Hip extension	17.1 ±5.1	17.6 ±5.8	16.1 ±4.4
• Hip abduction	50.4 ±8.7	51.4 ±8.5	48.7 ±8.6
• Knee flexion	123.4 ±14.9	124.7 ±13.1	124.2 ±11.6
• Ankle dorsiflexion knee extended	37.9 ±8.1	37.8 ±7.2	37.7 ±8.2
• Ankle dorsiflexion knee flexed	38.4 ±6.6	39.2 ±7.3	37.1 ±6.4

^Τ^: Normalized to limb length expressed as a percentage;

s: seconds; cm: centimetre;

°: degrees;

H: hamstring;

Q: quadriceps;

CONV: conventional;

FUNCT: functional

**Fig 2:**
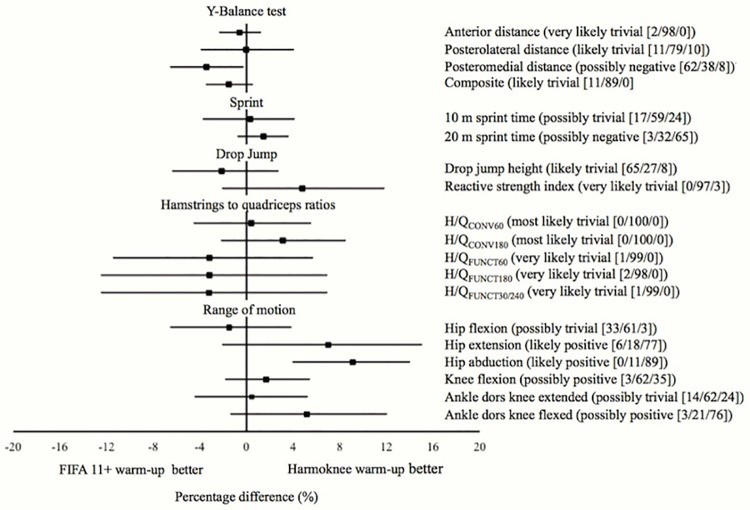
Net effects (expressed as percentage) of the interventions (paired comparisons) on the physical performance measures analysed for the FIFA 11+ and Harmoknee. The probabilities of an effect being harmful/trivial/beneficial are expressed as percentage values. Clinical inference is provided.

**Fig 3:**
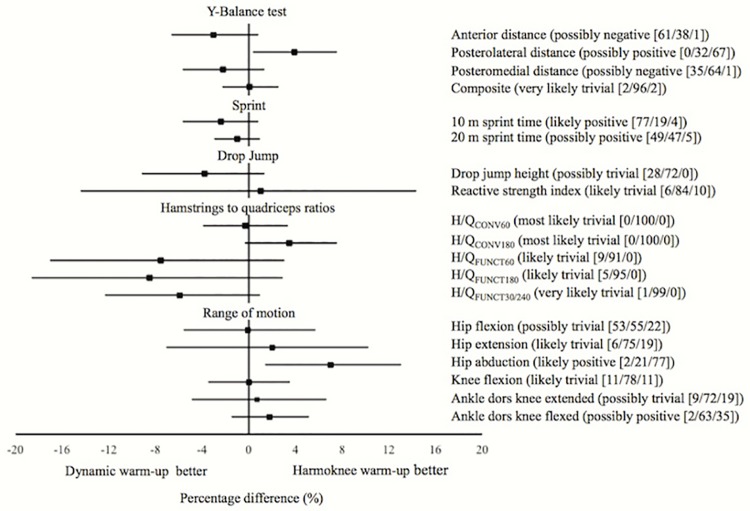
Net effects (expressed as percentage) of the interventions (paired comparisons) on the physical performance measures analysed for the Dynamic warm-up and Harmoknee. The probabilities of an effect being harmful/trivial/beneficial are expressed as percentage values. Clinical inference is provided.

**Fig 4:**
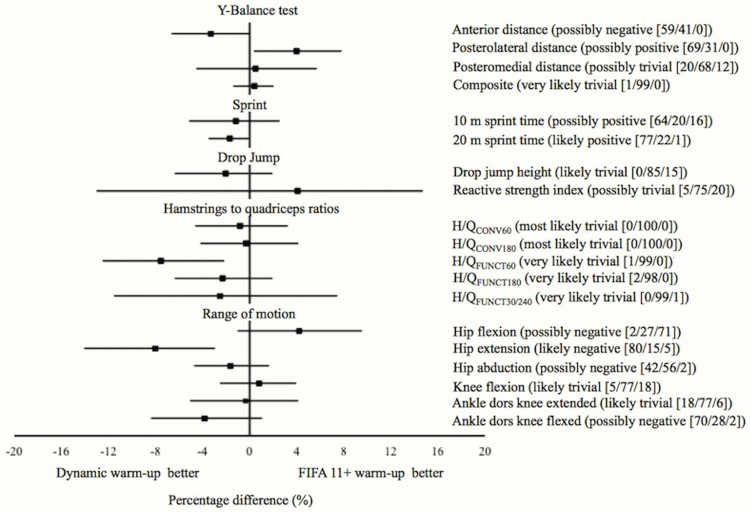
Net effects (expressed as percentage) of the interventions (paired comparisons) on the physical performance measures analysed for the Dynamic warm-up and FIFA 11+. The probabilities of an effect being harmful/trivial/beneficial are expressed as percentage values. Clinical inference is provided.

The paired inter-interventions percentage differences with the corresponding 90% confidence interval for the different physical performance measures are displayed in Fig 2 (FIFA 11+ warm-up vs. Harmoknee warm-up), Fig 3 (Dynamic warm-up vs. Harmoknee warm-up) and Fig 4 (FIFA 11+ warm-up vs. dynamic warm-up). No substantial differences (likely differences with a probability > 75%) were found between paired-comparisons for most of the physical performance measures. Only substantial differences (with a probability of 77%) were found for the sprint time outcomes, so that the FIFA 11+ and Harmoknee warm-ups resulted in slower sprint times in comparison with the dynamic-warm up for 20m (2.4%) and 10m (1.7%) respectively.

## Discussion

The primary findings of the current study reported that the acute (post-exercise) effects elicited by both the FIFA 11+ and Harmoknee warm-ups on most physical performance measures (with the exception of the sprint times) were similar (not substantial: probabilities of worthwhile differences <75%) to those found by the standard football-related dynamic warm-up routine. However, it should be noted that although not substantial and with a practical significance that is unclear, there appears to exist a tendency showing that the Harmoknee elicits superior improvements in the ROM measures than the FIFA 11+ (Fig 1). Perhaps a probable explanation for this positive tendency in ROM improvements in favour of the Harmoknee may be due to the fact that the Harmoknee, in contrast to the FIFA 11+, includes a specific element of stretching exercises (part number two: muscle activation). On the other hand, it should also be noted that another tendency appears to exist showing that the standard dynamic warm-up routine elicits higher improvements in the H/Q_FUNC_ ratios compared to those reported by both the FIFA 11+ and Harmoknee. Although speculative and based on the subjective perception verbally expressed by the participants, the specific eccentric exercise present in both the FIFA 11+ and Harmoknee, the Nordic curl, may have produced a certain degree of fatigue in this sample of players who were not used to performing eccentric exercises in their daily training sessions. As this is the first study (to the authors’ knowledge) to explore the post-exercise effects of the FIFA 11+, Harmoknee and dynamic warm-up routines on joint ROM measures, dynamic postural control and conventional and functional H/Q ratios, we are not able to make direct comparisons. Consequently, until future studies address this issue, the above-mentioned tendencies should be taken with a high degree of caution.

The current study also found that the standard dynamic warm-up routine elicited superior improvements in sprint times (1.7 and 2.4% for 10 and 20 m sprint times, respectively) when they were compared to those produced by the FIFA 11+ and Harmoknee routines (Figs 2 and 3). These findings are not in agreement with the results reported by Bizzini et al. [15], who found that the magnitude of the effects elicited by the FIFA 11+ on sprint times (2.2%) were comparable with those reported in the literature for dynamic warm-up routines (≈1.8%). A possible explanation for this discrepancy may be attributed to the different research design used in each study; Bizzini et al. [15] carried out a meta-analysis to compare the effects elicited by the FIFA 11+ with other warm-up routines previously published regarding football players, while we directly compared the effects elicited by the FIFA 11+ with a standard football-related dynamic warm-up routine. The higher positive effects in the sprint and jump height measures reported by the dynamic warm-up routine in comparison with the FIFA 11+ and Harmoknee routines might be partially due to enhanced activation of the history-dependent neuromuscular factors such as post-activation potentiation (PAP) and stretch-shortening cycle (SSC), which have been found mainly after isometric or resistance/weight-based exercises completed immediately before the task [24, 50–52]. Thus, the higher stimuli of resistance-based exercises presented in the exercises belonging to the final part of the dynamic warm-up routine (vertical and countermovement jumps, spiderman walks and sideways low squat walks) may have lead to a higher post-activation potentiation in comparision to the FIFA 11+ and Harmoknee. In addition, the previously mentioned possible presence of muscle fatigue as a consequence of the specific and novel eccentric exercise (i.e. Nordic curl) for the participants in both the FIFA 11+ and Harmoknee routines may also have contributed to this finding.

Although the current study is novel in several aspects (testing procedures, statistical analyses and design), some limitations should be noted. One limitation was the small sample size used in each group (interventions or controls). However, the sample size that was enrolled in each group was similar to previous warm-up studies [15, 18–22, 24, 27, 29, 30] and allowed main effects to be found. Another possible limitation of the current study is the sampling frame. The age distribution of participants (19.1 ± 1.3 y) and their physical skills level (amateur) were narrow and so generalizability cannot be ascertained.

## Practical Applications

The main findings of the current study reported that the three interventions (FIFA 11+, Harmoknee and standard dynamic warm-up routines) elicited similar acute (post-exercise) effects on several physical performance measures. However, superior positive acute effects on sprint measures were found in favour of the dynamic warm-up routine compared to both the FIFA 11+ and Harmoknee routines. Therefore, and based on the above-mentioned findings, neither the FIFA 11+ nor the Harmoknee routines appear superior to the dynamic warm-up routines currently performed by most football players prior to training sessions and matches. However, the FIFA 11+ and Harmoknee should be implemented in everyday football sessions as training components (i.e. placed in the main part of the training sessions), based on their demonstrated superior positive effects on injury prevalence rates in comparison with traditional practices.

## Supporting Information

S1 Appendix The passive hip flexion (passive straight leg raise test [figure a]), extension (Thomas test [figure b]) and abduction (passive hip abduction with knee flexed over the edge of the plinth test [figure c]); knee flexion (Modified Thomas test [figure d]) and ankle dorsiflexion (weight-bearing lunge with knee extended [figure e] and flexed [figure f] tests) range of motions assessment. (TIFF)

S2 Appendix Isokinetic testing position. (TIFF)

S3 Appendix Dynamic postural control assessment (anterior [figure a], posteromedial [figure b] and posterolateral [figure c] directions). (TIFF)
