# Supplementary figures and images for: Acute Effects of Three Neuromuscular Warm-Up Strategies on Several Physical Performance Measures in Football Players
Source: PLoS One. 2017 Jan 6;12(1):e0169660. doi: 10.1371/journal.pone.0169660 (PMC5218464; doi:10.1371/journal.pone.0169660)

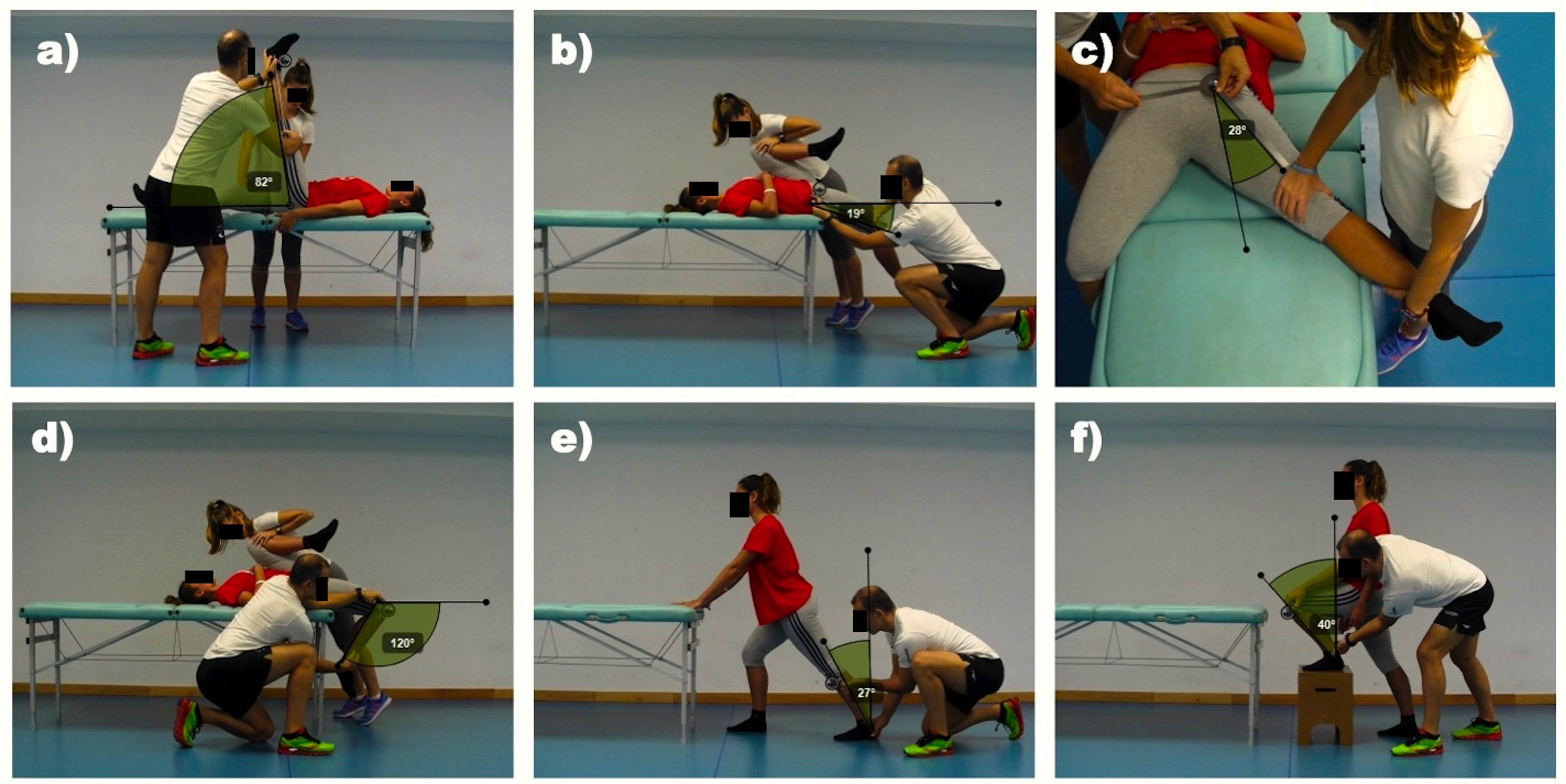

Supplement: S1 Appendix — (TIFF) [file pone.0169660.s001.tiff]

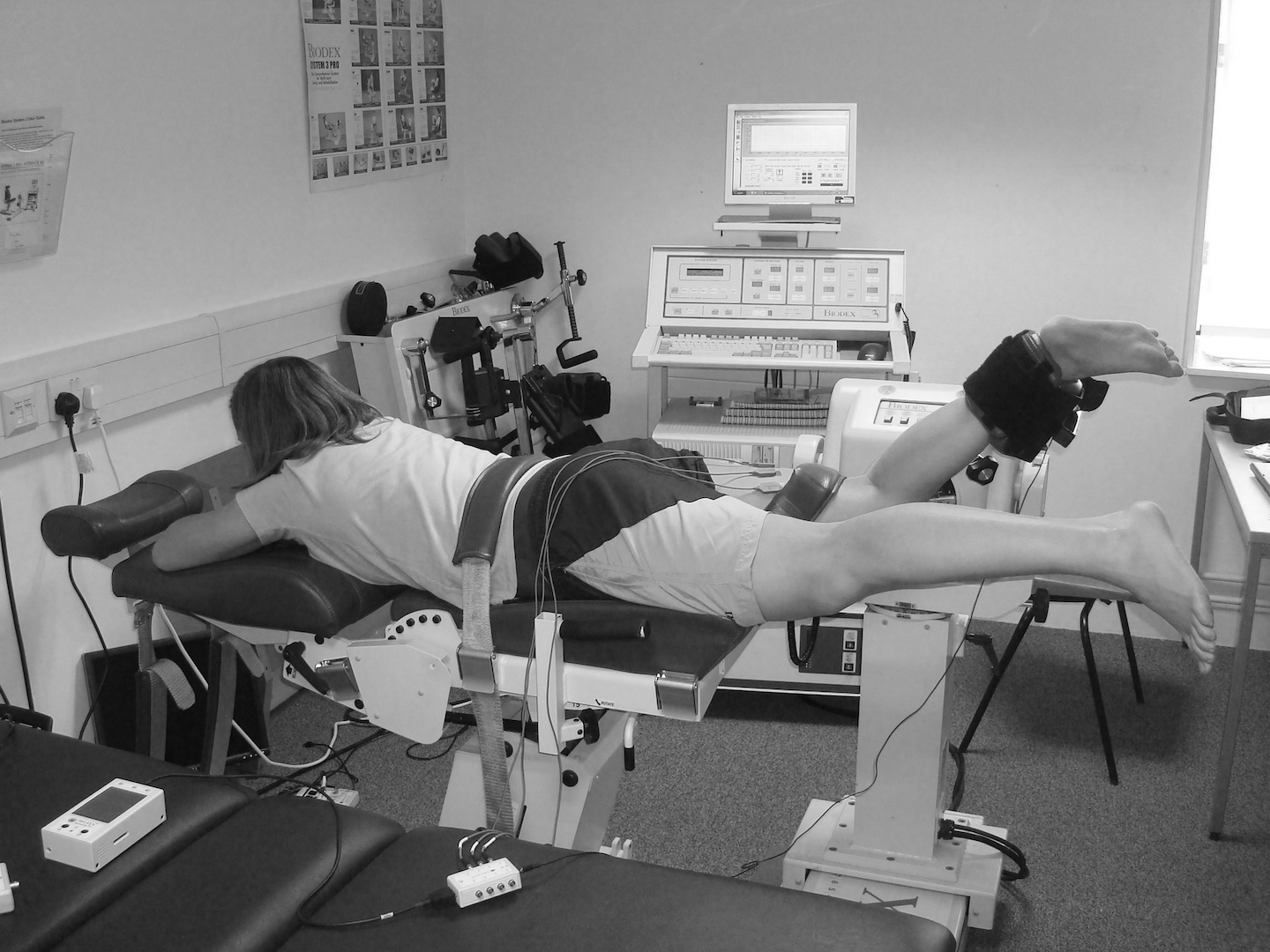

Supplement: S2 Appendix — (TIFF) [file pone.0169660.s002.tiff]

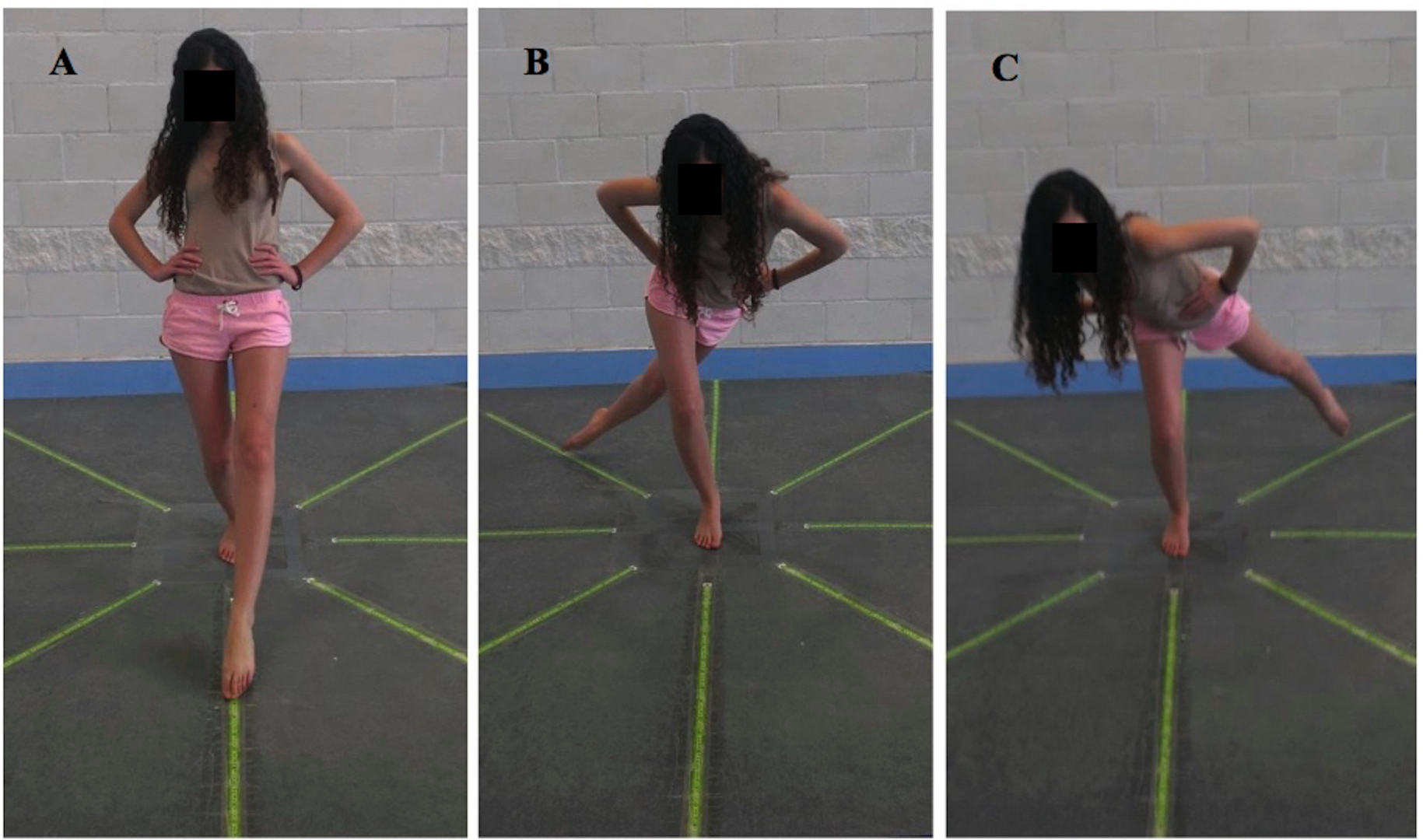

Supplement: S3 Appendix — (TIFF) [file pone.0169660.s003.tiff]
